# Introducing a depression-like syndrome for translational neuropsychiatry: a plea for taxonomical validity and improved comparability between humans and mice

**DOI:** 10.1038/s41380-022-01762-w

**Published:** 2022-09-14

**Authors:** Iven-Alex von Mücke-Heim, Lidia Urbina-Treviño, Joeri Bordes, Clemens Ries, Mathias V. Schmidt, Jan M. Deussing

**Affiliations:** 1 Max Planck Institute of Psychiatry, Molecular Neurogenetics, Munich, Germany; 2 Department of Translational Research, Max Planck Institute of Psychiatry, Munich, Germany; 3 International Max Planck Research School for Translational Psychiatry, Munich, Germany; 4 Max Planck Institute of Psychiatry, Neurobiology of Stress Resilience, Munich, Germany

**Keywords:** Neuroscience, Depression

## Abstract

Depressive disorders are the most burdensome psychiatric disorders worldwide. Although huge efforts have been made to advance treatment, outcomes remain unsatisfactory. Many factors contribute to this gridlock including suboptimal animal models. Especially limited study comparability and replicability due to imprecise terminology concerning depressive-like states are major problems. To overcome these issues, new approaches are needed. Here, we introduce a taxonomical concept for modelling depression in laboratory mice, which we call depression-like syndrome (DLS). It hinges on growing evidence suggesting that mice possess advanced socioemotional abilities and can display non-random symptom patterns indicative of an evolutionary conserved disorder-like phenotype. The DLS approach uses a combined heuristic method based on clinical depression criteria and the Research Domain Criteria to provide a biobehavioural reference syndrome for preclinical rodent models of depression. The DLS criteria are based on available, species-specific evidence and are as follows: (I) minimum duration of phenotype, (II) significant sociofunctional impairment, (III) core biological features, (IV) necessary depressive-like symptoms. To assess DLS presence and severity, we have designed an algorithm to ensure statistical and biological relevance of findings. The algorithm uses a minimum combined threshold for statistical significance and effect size (*p* value ≤ 0.05 plus moderate effect size) for each DLS criterion. Taken together, the DLS is a novel, biologically founded, and species-specific minimum threshold approach. Its long-term objective is to gradually develop into an inter-model validation standard and microframework to improve phenotyping methodology in translational research.

## Introduction

Depressive disorders are the second most common, yet most burdensome psychiatric disorders worldwide with regards to years lived with disability [1]. They are responsible for huge socioeconomic and personal costs and associated with increased morbidity and mortality [2, 3]. Trend analyses indicate an increasing prevalence, highlighting its growing global public health burden [4]. While our understanding of biological, clinical, and imaging features and biomarkers is mounting [5], and therapies have improved [6, 7], clinical outcomes remain heterogenous and vary depending on the individual and therapy modality [8, 9]. In fact, only 50–70% of patients recover within one year of diagnosis, while up to 40% experience multiple depressive episodes and 15% suffer a chronic course of disease [10–13].

A recent meta-analysis of 522 double-blind, randomized placebo-controlled trials confirmed the efficacy of 21 common antidepressants and their superiority over placebo [14]. Nevertheless, beside the serendipitous discovery of ketamine and brexanalone, the last few decades of research have not provided any novel and effective antidepressants [15–17]. Many factors contribute to this gridlock, including suboptimal animal models, poor clinical trial design and outdated drug approval processes [17–19]. Ever since the first animal models of mental disorders emerged, they have triggered considerable debate about their value and validity [20]. The frequent lack of reproducibility and context-dependency of data derived from animal experiments, especially in preclinical neuropsychiatric research, are issues that remain unsolved [21–24]. Despite the shortcomings and criticism, the importance of animal models in the fields of neurobiology and psychiatry cannot be denied [16, 20]. However, to move past current limitations and potentiate the impact of animal model-derived data on the psychiatric diagnostic and therapeutic progress, methodological improvements are urgently needed [15]. The obvious discrepancy between an increasing public health need, limited therapeutic dependability and suboptimal preclinical modelling demands critical evaluation of the way depression is conceptualized in clinical, translational, and basic neuropsychiatric research. Foremost, a shared and reliable framework for clinical and preclinical data is necessary. Advances should include innovative, replicable in vivo models and employ a precise and universal terminology concerning depressive-like states in animals [7, 15, 16, 25]. Here, we propose a methodological approach to address this issue for preclinical mouse models in translational neuropsychiatry.

First, we outline the depression criteria of the Diagnostic and Statistical Manual of Mental Disorders 5th version (DSM-5) and in the International Classification of Diseases 11th revision (ICD-11), discuss conceptual pros and cons and present emerging alternative taxonomical approaches. Second, we critically analyze the current use of the term ‘depressive-like’ in contemporary translational research in neuropsychiatry. Third, we propose a novel taxonomical approach for depression and its modelling, which we denominate as *depression-like syndrome* (DLS) in laboratory mice (*Mus musculus*). Here, we focus on mice due of their dominant role in translational psychiatric research and behavioural neuroscience, even though the approach per se is also applicable in other rodent species like rats. DLS is a threshold-based syndrome definition, hinging on a combinatory DSM-5/ICD-11 and Research Domain Criteria (RDoC) approach. We examine the potential of DLS as a novel consensus in the field of neuropsychiatry, capable of increasing comparability between models as well as overall face and construct validity. Finally, we devise a blueprint for DLS assessment and describe how it can be integrated into the existing scientific framework.

## Depression in the DSM-5 and ICD-11

The nosological entity labelled depression has been around since ancient times [26, 27]. Contemporary clinical taxonomy of mental disorders, as seen in the DSM-5 and ICD-11, primarily follows a symptom-based approach (Table 1). The classifications define psychiatric entities by appearance of select, yet only partially disease-specific phenomenological criteria (i.e., symptoms) over a certain period. These definitions often incorporate exclusion stipulations to allow discrimination between entities with a shared symptomatology. Conceptually, the classifications are similar, yet depression criteria differ slightly [28–30].

**Table 1:** Diagnostic criteria of a depressive episode in the DSM-5 and ICD-11.

Classification - syndrome definition		Diagnostic requirements	Severity assessment	Other
**DSM-5** Single depressive episode (296.21 ff.)	Criteria: (1) depressed mood, (2) diminished interest or pleasure in activities, (3) significant weight or appetite change, (4) insomnia or hypersomnia, (5) psychomotor agitation or retardation, (6) low energy/fatigue, (7) feelings of worthlessness or inappropriate guilt, (8) reduced ability to concentrate or indecisiveness, (9) thoughts of death, suicidal ideation, and/or suicide attempts	Five or more symptoms for ≥ 2 weeks including (1) and/or (2)	Based on symptom amount and intensity and functional impairment (mild, moderate, severe).	'Specifiers’ to represent clinical depression heterogeneity
**ICD-11** Single episode; depressive disorder (6A70 ff.)	Criteria: (1) depressed mood, (2) diminished interest or pleasure in activities, (3) low concentration, indecisiveness, (4) feelings of low self-worth, (5) hopelessness towards the future, (6) thoughts of death, suicidal ideation, and/or suicide attempts, (7) disturbed sleep pattern, (8) changes in appetite or weight, (9) psychomotor agitation or retardation, (10) low energy/fatigue	Significant distress or impairment in occupational, social, or other important areas of functioning.	Based on overall symptom intensity and functional impairment (mild, moderate, severe).	'Qualifiers’ to represent clinical depression heterogeneity

The DSM-5 defines depression as daily presence of five out of nine possible symptoms for a minimum of two weeks, for nearly all the time. One symptom must be depressed mood or strongly diminished interest/pleasure in daily activities. Disease severity is determined by overall symptom number, individual distress and social as well as occupational functional impairment. In addition, the symptoms should not be better explained by physiological effects of a substance or another medical condition [29, 31–33]. To assess functional impairment, the Global Assessment of Functioning Scale (GAF) has been developed based on the DSM criteria. It is a severity estimate score of psychiatric conditions ranging from 0 to 100, which accounts for overall symptom severity and functional impairment [34, 35].

The more recently updated ICD-11, on the other hand, defines a depressive episode by daily occurrence of five out of ten symptoms for a minimum of two weeks. The symptoms are similar to the DSM-5, except for the additional symptom hopelessness, which was included because of its reliability to discriminate between depressed and healthy subjects [28, 36]. As with the DSM-5, one symptom must be depressed mood or strongly diminished interest or pleasure in daily activities. Analogous to the DSM-5 exclusion stipulation, the symptoms should not be better explained by the effects of a substance, other medical condition, or bereavement. Disease severity is based on symptom intensity and overall functional impairment. In comparison to earlier ICD editions, the latest edition provides better representation of symptom heterogeneity, global and clinical applicability as well as an improved discrimination of diseases with overlapping symptomatology [28, 32, 37–39].

Overall, the ICD-11 depression criteria and severity estimates are closely related to the DSM-5 [28, 29]. The revision has aided the alignment of the two major classification systems and is therefore meeting the call for conceptual consistency in mental health taxonomy [40]. Due to this similarity, the ICD-11 and DSM-5 share most of their strengths and weaknesses in the context of depression [16, 28, 41, 42].

## Novel alternatives to established clinical taxonomies

Although expert-consensus classifications like the DSM-5 and ICD-11 are an integral part of current clinical practice, they have triggered considerable debate [40, 43–46]. For the DSM-5, some of the more disputed features are its weak scientific foundation, arbitrary disease definitions, categorical disease approach and the associated loss of clinical data, insufficient representation of within-diagnosis heterogeneity, limited reliability and validity, negligence of frequent comorbidities, failure in establishing diagnosis-selective treatments, undependable severity assessments and predilection for false positive diagnosis [31, 40, 41, 47–50]. In contemporary clinical and scientific practice, the boundary between mental health and disease is arbitrary. Categorical distinctions are based on vague symptom clusters, which were established based on historical, and now debatable, empirical pillars [40, 41, 43, 46–48].

Given the limitations of the DSM-5 and ICD-11 [51], novel taxonomical approaches attempt to reduce arbitrariness and uncertainty in neuropsychiatry. For example, the ongoing BeCOME study uses deep phenotyping and a multi-omics approach to establish a biologically grounded classification of mental disorders [52]. Meanwhile, the Hierarchical Taxonomy of Psychopathology (HiTOP) follows an empirical bottom-up approach and defines psychiatric entities according to covariation of psychopathological symptoms, quantitively and based on available evidence. It proverbially aims to overhaul the DSM-5 to improve reliability, validity, and clinical utility [47, 48, 53]. Even though the HiTOP and BeCOME study are just two examples, they epitomize the field’s recent efforts to advance clinical taxonomy towards a more reliable and valid approach, both on a phenomenological and biological level.

## Depressive-like behaviour and the Research Domain Criteria

Animal models of depressive disorders are mostly based on the induction of biological or behavioural changes seen in depressed patients using a psychosocial or biophysical stressor. Traditionally, rodent models have been judged based on face, construct, and predictive validity [17, 54]. Behavioural measures in laboratory mice include, but are not limited to, anhedonia, apathy, anxiety, despair and hopelessness, irritability, social aversion, cognitive impairment and disturbance of feeding, sleep, and psychomotor activity. These behaviours, either one alone or in an undefined combination, are commonly considered depressive-like phenotypes [7, 17, 55, 56]. The rationale behind this is that non-human animals including mice are commonly considered unable to experience depression in its phenomenological and biological entirety, yet they display behaviours similar to depressive symptomatology in humans. These behaviours are induced by causative factors (e.g., environmental, genetic), can be quantified by standardized behavioural tests and should respond to established antidepressants. Standard assessments include the forced swim test, tail suspension test and sucrose preference test. The latter is deemed the equivalent of anhedonia and is thus considered at the core of the depressive-like spectrum in laboratory mice [7, 15–17, 20, 25, 57, 58]. However, in contrast to the clinical setting, there is no consensus or universal standard regarding the composition and duration of the observable behavioural phenomena to be termed depressive-like in laboratory mice. Rather, any behaviour that is part of the depressive symptom spectrum is called depressive-like in mouse models, mostly independent from the induction scheme, its severity, composition, or duration [7, 16, 17, 55, 59–62].

The hypothesis that depressive behaviours are somewhat comparable between species, here humans and mice, rests on the shared phylogenetic origin and the subsequent interspecies conservation of biological and neuroanatomical structures necessary for complex emotions, cognition and behaviours [57]. The latter must be considered the *conditio sine quam non* for psychiatric disorders. In line with this assumption, the National Institute of Mental Health has developed the RDoC. It is a comprehensive framework for psychobiological research comprised out of five domains, which represent categories of interspecies homologies on a phenomenological, construct and network level. These domains are positive and negative valence, arousal and regulation, social processes, and cognition [42, 57, 63, 64]. Recently, the sensorimotor systems domain was added [65, 66]. Overall, the RDoC matrix is an elaborate and multidimensional tool, which enables an empirically substantiated default matrix of comparison and two-way translation between humans and laboratory rodents [42, 57, 67]. The RDoC enterprise is a work in progress and has been termed a “calculated gamble” by Lilienfeld [44]. It has been criticized for being reductionistic and inadequate in important clinical disease features [44, 45, 67, 68]. Furthermore, since the RDoC is neither a clinical classification nor established beyond reasonable doubt, it still needs to prove its scientific and societal value [42, 67, 69–71].

Combining the symptom-based approach of DSM/ICD with the evolutionary conception of the RDoC yields great potential in the field of translational neuropsychiatry. Merging the two concepts may result in a revolutionary, integrative, and accurate classification which could balance the inherent advantages and drawbacks of the different approaches [67, 72, 73]. This is critically important since precise and integrative rodent models of depression are still lacking [15, 16] and comparability and reproducibility, especially of behavioural paradigms carried out in laboratory rodents, is in great need of improvement [21–24].

To refine mouse models of depression with a clinical research aim, we suggest revisiting the use of the established yet undefined term ‘depressive-like’ considering the integrative and combinatory heuristic approach. Simultaneously, we advocate an ongoing dialogue between clinicians and neuroscientists to advance back-forward-translation, scientific discovery, and eventually improve therapies [7]. Here, we argue that a combined DSM/ICD/RDoC-definition of ‘depressive-like’ behaviour in the form of a murine DLS could help produce replicable and comparable animal studies, improving overall validity and clinical impact.

## Arguments for a standardized depression-like syndrome in laboratory mice

To advance towards a combination of the phenomenological DSM/ICD and the neurobiological RDoC in translational depression modelling, we argue that a novel approach needs to merge core depression symptoms, interspecies commonalities and clinical as well as biological heterogeneity, framed by evolutionary conserved, species-selective considerations.

Based on the syndromic nature of depression entrenched in the DSM/ICD, the behavioural and biological interspecies homologies and evolutionary assumptions of the RDoC [42, 57, 63, 73], and the cogent evidence from human and animal studies unveiling the behavioural and biological mechanisms of chronic stress and depression, it can be reasoned that certain features of depression neurobiology and symptomatology are conserved and thus shared between related species including humans and mice, while others are not [6, 16, 17, 74–78]. For example, the DSM/ICD criterion of inappropriate guilt entails two presuppositions: the concept of inappropriateness and of guilt. Both are uniquely human and thus species-specific concepts. Guilt behaviour in dogs could not be proven [79–82] and no tests of guilt have been advocated or used in rodents in the context of depressive-like behaviour. The same line of argument stands for suicidal ideation and suicide attempts [83]. For most of the other DSM-5 depression criteria, Czéh et al. have provided a detailed and up-to-date matrix linking them to observable physiological and behavioural domains and available tests in mice [83].

In clinical practice, diagnosis is based on self-reported symptoms and the psychopathological evaluation by a health care professional, which is then matched with diagnostic criteria of the DSM/ICD [84]. However, it is common practice to base response and remission calculations in clinical depression studies considerably or exclusively on clinical self-rating instruments like the Patient Health Questionnaire (PHQ-9) or the Beck-Depression-Inventory (BDI-II) [85–87]. This approach focuses on real-world feasibility by condensing depression complexity into a short questionnaire, which solely reflects an internal perspective. While this practice has its strengths and weaknesses, it sets a standard approach for measuring symptom burden in clinical practice and research. Nevertheless, the PHQ-9 has shown good validity and reliability as well as adaptability to diagnose depression [88]. Based on this clinical approach and in line with the notion of between-study comparability, interspecies homology, and the idea of a transspecies translational matrix for DSM/ICD depression criteria we argue that a similar standardized approach can be devised for laboratory mice. This approach should be empirically validated and progressively improved for in vivo depression models.

Mammals share parts of their evolutionary and phylogenetic path [44, 57], as well as the environmental and social stress during development [89–91]. Furthermore, both mice and humans exhibit so-called *sickness behaviour*—an evolutionary conserved biobehavioural response to infection or another immune trauma—with symptoms overlapping depressive symptomatology [92]. Relating the current standards to the paradigms and behavioural tests used to induce and measure depressive-like behaviour in mice [16, 17] it can be reasoned that related species are predestined to develop similar but not necessarily identical symptoms indicative of a shared emotional and cognitive continuum. Growing evidence supports the advanced abilities of non-human mammals like mice [93, 94] including, but not limited to the notion of self-awareness, consciousness [95–99], cognition [100, 101], personality-like traits [102, 103], and complex social capabilities [93, 104], along with the ability to suffer [105] and experience emotions [101, 104, 106, 107]. Aside from ongoing debate concerning theory of mind in non-human mammals and the uniqueness of language [108, 109], we argue that certain non-human mammals including laboratory mice meet the minimal requirements of an ethologically ascertainable depressive-like state, namely typical neurobiological and socioemotional features. This agrees with the notion that the core experience of depression is that of a sustained negative affective and emotional state (i.e., sadness and anhedonia), which is accompanied by a subsequent and non-random socioemotional symptomatology [110]. This perspective shares the ‘if-then’ logic of the core and side symptom concept of the DSM/ICD [28, 29, 31, 32, 36]. Ethological studies have proposed a faunal equivalent of posttraumatic stress disorder and clinical depression in Asian elephants [111–114], chimpanzees [115, 116] and macaques [117]. These studies indicate that non-human mammals can display non-random and timewise stable symptom patterns indicative of a disorder-like neuropsychiatric phenotype. Ultimately, this similarity between human and certain non-human mammals suggests the notion of applying clinical, human-specific criteria of a stress-related syndrome like depression onto mice to be valid, however to a currently still uncharted, undefined, and non-standardized extent. Based on the presented evidence and the need for novel models in neuropsychiatric research [15], we ask the scientific community to consider mice as being able to display a socioemotional syndromic state related to clinical depression, virtually a species-selective faunal depression-like syndrome. We thus suggest to collectively work towards an evidence-based standard definition of this state to enhance interspecies and inter-model homology, construct, and face validity along with comparability and generalizability of findings. We term this notional faunal equivalent a DLS.

Currently, one hypothesis of a clinical criteria-based depressive syndrome equivalent in mice has been proposed. It rests on the link between chronic stress protocols and behavioural patterns in mice related to depression. Dzirasa and Covington 2012 developed a hypothesis, which they named a *mouse affective syndrome* (MAS) [118]. In line with the recommendations of Nestler and Hyman 2010 [119], the authors state that chronic stress regimens lead to an ethologically delimitable syndrome in mice, which represents the core features of depression. They propose using clinical criteria to assess murine behaviours framed by three larger biobehavioural domains: (i) reward related, (ii) homoeostatic factors, and (iii) biomarkers. The clinical, symptom-based approach is merged with a biological framework to classify a depressive-like state in mice. Although the MAS has not yet gained mass attention, we believe the underlying hypotheses and approach to be promising.

## Blueprint of a murine depression-like syndrome

In the following, we draft the blueprint of a murine DLS. Analogous to the DSM/ICD criteria, the DLS aims to define a stable, multimodal, and primarily phenotype-based read-out rather than the depiction of a phenotypes’ inducibility. Nonetheless, aside the read-outs of interest, the induction method applied in a particular model should be provided to enable the evaluation of aetiological validity [16]. The latter is particularly important, since often used techniques to induce stress and model depression-like chronic social defeat have been originally designed for male mice [120, 121]. Just recently, protocols feasible for females have been devised [122, 123]. Analogously, certain behavioural assessments like the urine sniffing test have only been established for male mice [124]. These sex-related constraints and idiosyncrasies need to be taken into consideration in a translational framework like the DLS, meaning that both male and female mice should be included in an experimental set up and that any measurement methods including behavioural assessments must account for this. This need is substantiated by the fact that depression affects females about two times more often than males [125], as well as by a recent study by Kang and colleagues, which has demonstrated a significant difference in the genetic architecture between depressed men and women [126].

The DLS read-outs are based on the DSM-5 and ICD-11 and define the minimum phenotypes necessary, which must be present over a defined period. These symptoms are assessed and quantified using established behavioural tests and assigned to neuroevolutionary conserved domains or subconstructs of the RDoC framework. However, due to the uniqueness of human language [109], “operationalization” is exclusively based on external ratings [83]. Moreover, symptoms need to be severe enough to cause significant suffering or sociofunctional impairment to qualify as a DLS. In line with the DSM-5 and ICD-11 approach, symptom severity and its impact on sociofunctional domains is used as a syndrome severity proxy [28, 32]. Analogous to the long-established approach of the GAF [34, 35], DLS severity considers the entire sociopsychological state and functional impairment of mice. The definition also entails core biological features of depression [17, 19, 56]. Taken together, the DLS reflects two of the core model criteria, which are face validity (DSM/ICD-based syndrome) and construct validity (major biological aberrations) [17, 54, 56, 127].

The symptoms of a DLS should ideally be uniform and, as far as possible, species independent to foster reciprocal human to mouse comparability and two-way translation. However, the biological and behavioural inter-individual and -species heterogeneity renders this approach fruitless [118, 119, 128–132]. Therefore, DLS read-outs and time criteria must be tailored to an individual species, here mice, based on current evidence. This way, the DLS definition can be formulated *ad interim*, which can then serve as a starting point for its empirical substantiation and improvement.

**Fig. 1:**
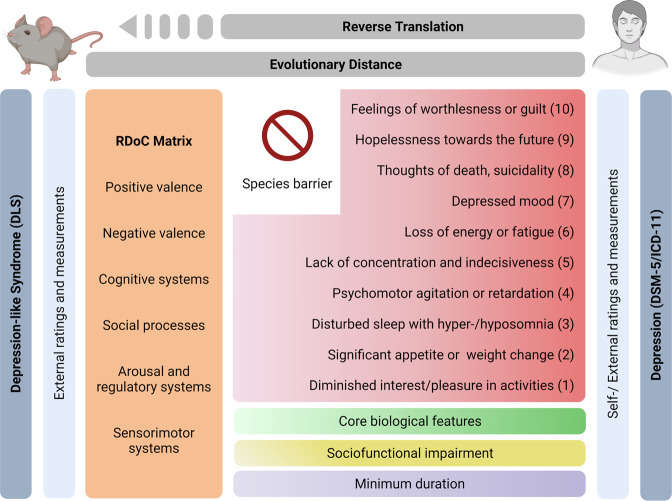
The depression-like translational matrix: from humans to mice, and back. Starting from the DSM/ICD-based depression definition, the translational matrix illustrates the process of reverse translation from clinical and thus human criteria to mice. While some of the depression symptoms (e.g., feelings of worthlessness) cannot be found in mice due to the evolutionary distance and respective species barrier, many others in addition to core biological features, sociofunctional impairment, and a minimum duration can be operationalized and quantified in mice (e.g., reduced appetite or significant weight loss). These read-outs, both in humans and mice, can be shelved into RDoC domains (Created with BioRender.com).

Below we outline DLS criteria specifically for laboratory mice due to their broad use in translational research. Criteria are outlined from general to specific. For illustrative purposes, we have created a translational matrix to highlight the different aspects of the translative process underlying the DLS hypothesis and the relation between mice and humans (Fig. 1).

### Minimum duration

In the DSM-5 and ICD-11, diagnosis requires 14 consecutive days with symptom presence almost all the time [28, 33]. In a real-world setting, depending on the extend of professional care provided, a major depressive episode on average lasts between 8.1 and 9.5 months [133]. Thus, it can be reasoned that an episode, on average, lasts 8-10 months. To translate this into laboratory mice, a between-species age-translation matrix is necessary. There have been a variety of attempts to relate the age of non-human mammals to the one of humans [134], for example using the eye lens weight [135], growth plate closure time [136], or molar ageing [137]. Based on the elaborate but practicable human-mouse-matrix developed by Dutta and Sengupta [134], which similar to the human-rat-matrix by Sengupta [138] is mainly based on developmental stages, 10 human months would translate into 52 h (2.16 days) in young adult mice (aged 10–64 weeks) and 176.5 h (7.35 days) in older, presenescent mice (aged 65–72 weeks). In direct contrast, translating the DSM/ICD’s 2-week criterion to mice is pointless, since this would amount to 2.4 h (0.1 days) and defy aetiological plausibility. After all, a day in the life of a mouse has the same total duration as a day in the life of a human. Still, recent studies demonstrate cellular and developmental processes of mice to be faster than those of humans [139–141]. Taken together, these findings highlight the complexity of age-translation between species [24] as well as the importance of age selection in mouse models [142]. To account for these developmental, cellular, and species differences, we would argue, that within the above-deducted range between 52 and 176.5 h, a longer rather than a shorter phenotype threshold is preferable. This way, the sociobiological state evoked is likely to impact the long-term individual condition of the respective mouse in terms of its reproductive success, social bonds, and physical fitness. We argue that using 52 ho of phenotype detection and presence as a minimum DLS duration definition is insufficient to significantly and reliably cause chronic, sociofunctional impairment in adult mice. But, this long-lasting sociofunctional impairment is a pivotal component of validity in the context of the DLS framework, since these ramifications essentially are the species analogue of the long-lasting socioeconomic and physical disadvantages faced by depressed patients [3]. Therefore, we recommend 7 days of phenotype presence as the DLS minimum duration. This increases the overall face validity of the DLS framework, in particular with regard to the time component of depression. However, since this analogical conclusion is primarily deduced from the human condition, it warrants step-by-step empirical adjustment so that the species differences depict the actual naturalistic duration of “murine depression” and the DLS

Due to the limitations of conventional behavioural tests, foremost the lack of repeatability of certain assessments, and the unavailability of self-rating, DLS duration needs to be captured by non-invasive methods like video tracking. This way, it is possible to ascertain which symptoms are continuously present. For this purpose, we recommend the use of continuous home-cage monitoring, as this reliably captures many depressive-like symptoms over time [143]. The feasibility of this approach using social boxes has been demonstrated many times, most recently by a work of Lopez and colleagues [144]. Still, since regular animal handling as well as other procedures and behavioural tests need to be performed during DLS assessment, continuous tracking appears difficult to some extent. More so, since handling and testing can influence home-cage activity and introduce significant bias. For the DLS framework, we thus recommend multiple short home-cage tracking periods spaced a few days apart of each other. Using this approach, the time component can be approximated via multiple measurement points of the same variables of interest, while bias can be minimized. Depending on the psychosocial or biophysical stressor used, serial measurements of social or functional impairment could serve as a proxy to ensure the evoked sociofunctional impairment phenotype is stable over the 7 days.

From a feasibility standpoint, proxies of DLS duration should primarily be based on more general symptoms like sociofunctional impairment, and only secondary on singular symptoms, like anhedonia. The rationale is that sociofunctional impairment can be measured non-invasively, whereas symptoms like anhedonia or impaired concentration and indecisiveness require hands-on assessments like the sucrose preference test or a novel object exploration task [17, 83, 145]. Thus, social or functional impairments could serve as proxies of syndrome duration, since they are relatively easy to measure, hardly bias the phenotype by investigator manipulation or enrichment, and can be combined with other depressive-like behaviours such as anhedonia [17].

Symptom duration cannot be translated directly into one distinct RDoC matrix domain, yet it can be co-reported with other factors. For example, increased duration of social avoidance can be integrated into the social process domain and changes of innate motor patterns could be assigned to the sensorimotor systems domain.

### Sociofunctional impairment

In laboratory rodents, social impairment can be assessed by a plethora of social interaction and avoidance paradigms [83]. These paradigms make use of video tracking methods to quantify parameters like time spent in the interaction or avoidance zones and total movement [120, 146]. Meanwhile, functional impairment in mice is a more complex affair. For face validity, we advocate to base murine functional impairment on the clinical approach, namely the inability or negligence to perform important tasks in different areas of daily life [28, 33]. Thus, we propose a reduction in goal-directed behaviour (apathy) to assess murine functional impairment, since it reflects self-neglect and the inability to fulfil a sociobiological role. Examples of apathic mouse behaviours are impaired nest building, deterioration of coat state, reduced self-grooming and maternal care as well as diminished social interest [17, 147, 148]. Although classical social interaction and avoidance paradigms still play an important role in preclinical behavioural research, they have several limitations including the dependence on the human observer and a heavy susceptibility to confounders [149]. For that reason, novel approaches are emerging, which enable continuous assessment of complex rodent social behaviours in a naturalistic setting. Nilsson et al., for instance, have developed an open-source analysis package for freely moving rodents called Simple Behavioural Analysis (SimBA) [149]. It uses pose-estimation and supervised machine learning to provide predictive classifiers for rodent social behaviour. Another related approach is the Social Box paradigm, which was recently successfully used by Forkosh et al. [150]. It captures behaviours of freely moving mice in a group and a semi-naturalistic setting over a period of days to depict individual differences using mathematical modelling.

Taken together, using traditional or video-based assessment of sociofunctional behaviours, murine impairment can be effectively operationalized and quantified. However, the specific psychosocial or biophysical stressor regimen used to evoke chronic stress-related phenotypes may influence or limit suitable assessment methods. These quantitative and qualitative results can, for the most part, be assigned to the social processes domain of the RDoC matrix.

### Biological features

To guarantee a minimum of construct validity, we propose to include pertinent and empirically substantiated biological factors of depression in the DLS definition and framework. This way, the problems associated with phenotype-based syndrome definitions like in the DSM-5 or ICD-11 are attenuated. The addition of biological read-outs respects the complex biobehavioural nature of depression. However, due the biological heterogeneity of depression [130, 151], and the plethora of aberrations associated with the disease [75], not one but several factors should be considered to determine the biological underpinnings of a DLS. Many studies have identified a complex network of biological factors, which jointly feed into the final common biological pathway of chronic stress and depression, that is a reduction in neuroplasticity in neurogenic regions [152, 153]. In particular, chronic stress and depression cause impaired neuroplasticity in the hippocampus and prefrontal cortex [74, 75, 152], which can be quantified by different methods, depending on the plasticity dimension (e.g., molecular, network or functional neuroplasticity) [152]. In murine models of depression, cellular and molecular neuroplasticity is often quantified by use of bromodeoxyuridine (BrdU) immunohistochemistry [154]. However, other biological parameters can also be used to substantiate a DLS, for example cortisol levels, neuroinflammatory markers like C-reactive protein, or adrenal gland weight. We recommend using robust and common disease features and to refrain from rare phenomena.

Impaired neuroplasticity, along with elevated cortisol levels or increased adrenal gland weight, could be included into the appropriate RDoC subconstruct (e.g., cells, molecules, or physiology) of the domain, in which the measured DLS symptom cluster belongs.

### Depressive-like symptoms

As set out previously, disease and syndrome definition in clinical psychiatry and the DSM/ICD are, though experience-driven, socially, and historically skewed, and thus arbitrary to some extent. To improve this partially evidence-based *status quo*, we propose a data-driven approach for minimum and core symptom determination. However, it must be noted, that aside from methodological rigour, a DLS neither can nor should fully comply with the clinical classification and symptoms of depression, but rather match the most common and core criteria. Analogous to the DSM/ICD symptom criteria that is the simultaneous presence of one core plus multiple additional, but depression-typical symptoms, we argue, that the DLS needs to resemble this clinical one-plus-phenotype and syndrome. In line with the neuroevolutionary arguments presented above, it is reasonable to assume a significant overlap between humans and mice. Nonetheless, one must account for the evolutionary and biopsychosocial differences between the two species, which separated about 75 million years ago [93]. This ambivalence in the context of a partially shared ancestry and somewhat related biopsychosocial capacities is important because the underlying hypothesis of a murine DLS is to capture the faunal equivalent of clinical depression: a murine depressive-like state reflective of an evolutionary conserved entity in certain higher non-human mammals. This idea of depression as an evolutionary explicable entity intertwined with evolutionary psychiatry and psychopathology has already been brought forward by many scholar [131, 155, 156].

In a clinical setting, anhedonia is reported in about 90% of majorly or severely depressed patients [32, 157] and is well suited to distinguish severe from moderate depression [32]. Additionally, the other core symptom, depressed mood, and sadness, is considered unique to humans [74, 83]. Based on this evidence and the DSM/ICD criteria, we advocate anhedonia as the core DSL symptom. Although it has been discussed, even proposed that animals are capable of emotions [101, 104, 107, 158], the core issue remains unsolved: in non-human mammals, observational and biological measurements are insufficient to determine whether data indicative of a certain emotion actually reflect the equivalent of human sadness [93, 106]. Since we cannot talk with a mouse to assess its inner state, the verification of sadness is impossible; at least for the time being. Nevertheless, following the line of argument of animal emotion presented above, it might be fair to assume a sadness equivalent or least sadness-related emotional experience in mice. In contrast, anhedonia is widely accepted in the scientific community in regards to murine depression models and can be readily assessed, for example by the sucrose preference or cookie test [17], intracranial self-stimulation techniques [159], conditional place preference, or urine sniffing [83]. Admittedly, using anhedonia as the DLS core symptom inevitably leads to a definition revolving around anhedonia-present depression. However, Buckner et al. 2008 were able to demonstrate that although anhedonia-present depression differs from sadness-present depression in terms of symptom cluster, there is an overlap in symptoms, particularly regarding reactivity of mood, social impairment, and social withdrawal [157]. Patients with a strong depressive phenotype (i.e., severe clinical depression) display a high concurrence of the DSM-5 core depression symptoms: 100% of patients report sadness, and 90% anhedonia [32]. Based on this, we would argue that a distinction between anhedonia- and sadness-present depression is mostly artificial and not representative of the real-world situation. Thus, centring the DLS definition around anhedonia is reasonable.

On these grounds, we propose to define the murine DLS symptom cluster centred around anhedonia, meaning that anhedonia plus additional, depression-typical symptoms need to be present to constitute the DLS. Concerning these additional, depression-typical symptoms, Czéh et al. have developed a detailed matrix that matches most DSM-5 depression criteria to distinct physiological and behavioural phenomena as well as available quantification methods in rodents [83]. This comprehensive matrix can serve as a basis for the assessment of DLS symptoms. But the question remains: how many additional symptoms are necessary for mice? Due to the species barrier and the fewer depressive symptoms present in mice, we advocate a total of three additional symptoms. To assess the presence of each depressive symptom, we suggest using two different tests, if possible. For instance, to confirm the presence of anhedonia, mice should display biologically plausible and robust differences to a respective control group in the sucrose preference and urine sniffing test. Using this approach, symptom presence is concurrently validated by two measurements of the same symptom, which increases validity and power and enables binarization. Ultimately, this allows to confirm the presence or absence of a symptom. The number of symptoms necessary to constitute a valid and robust depressive-like phenotype and DLS remains to be determined. Prospectively, this can be achieved using a fact-driven, prospective clustering approach, that consolidates the association between different symptom clusters, the other DLS criteria including anhedonia and other, already validated, murine depression models.

Due to the versatile quality of depressive symptoms (cognitive, behavioural, emotional, and somatic), the respective symptom can be easily translated to the RDoC matrix. Impaired concentration and indecisiveness, which can be measured using a Y-maze or a novel object exploration test [83], belong in the RDoC cognition domain. Meanwhile, a disturbed sleep pattern is part of the arousal and regulation domain. Similarly, most DSM-5 symptoms can be categorized into a specific RDoC domain.

### Syndrome severity

As outlined above, the DSM-5 estimates depression severity on symptom quantity and intensity, and social as well as functional impairment [29, 31, 33]. In a clinical setting, these criteria are assessed by instruments such as the Hamilton Depression Rating Scale (HAM-D), BDI-II, or GAF score [32, 34, 35]. Similarly, DLS severity estimates should be based on overall sociofunctional impairment and symptom degree [28, 32]. A recent study by Elmer and Stadtfeld 2020 on the *depression-isolation hypothesis* found that depressed individuals spend less time in social interactions and with known friends, and more time with depressed others and in pair-wise interactions. In addition, the study found that depressive symptoms were negatively correlated with the time spent interacting socially [160]. These findings support the notion that a reduction or non-beneficial change of social behaviours can serve as the primary DLS severity proxy. In addition, symptom degree could be added to approximate the DSM-5 outline. However, in contrast to the DSM-5 and ICD-11 severity assessment, which lacks quantitative standardization, we would argue that to achieve a reliable and biologically valid severity estimate, the approach should be strictly data-driven and based on indexing sociofunctional impairment and co-occurring symptoms. Fortunately, the available tests enable quantification and subsequent binarization of symptoms [83]. Whether the clinical severity categories ‘mild, moderate, and severe’ are detectable and useful in mice remains to be empirically determined [33].

## Statistical algorithm for depression-like syndrome assessment

Having outlined the DLS blueprint and framework, we finally propose an algorithm to assess the presence of a DLS and its severity. This algorithm uses statistical instruments to guarantee a standardized and data-driven approach.

Due to the issues arising from the difference between statistical and biological significance or relevance, and the associated risk of false discovery [161, 162], we argue that basing DLS criteria presence on statistical significance alone is insufficient. We suggest the combined use of effect size and statistical significance. Given the principal validity of the biobehavioural DLS framework, this dual heuristic approach would enable the biological significance and plausibility of a difference between a group of interest and healthy controls to be found with high probability [163, 164]. This hybrid concept has been termed a *minimum effect size plus p value* (MESP) by Goodman et al. [163]. For the DLS, we recommend a statistical significance level of *alpha* ≤ 0.05 as well as a moderate effect size (for example: Cohens *d* ≥ 0,5 or Cohens *f*^2^ ≥ 0.25^2^, depending on the respective statistical measure) as the minimum combined threshold. These DLS-MESP criteria should be obtained from comparison between the group of interest and a healthy control group using sample-appropriate statistical testing and, if necessary, post-hoc correction. We suggest employing two appropriate read-outs for each DLS criterion. Tests and read-outs should meet the DLS-MESP criteria. If only one of the two selected read-outs per symptom domain meets the DLS-MESP criterion, it might be reasonable to report the respective finding and base binarization (DLS symptom present: yes/no) on overall biological plausibility by considering other data for the same DLS criterium and animal. In select cases, however, it might also be feasible to augment a third measurement for the symptom and DLS criterium in question to substantiate binarization in the case of inconsistency between the initially selected two read-outs. Alternatively, investigators might consider assessing another DLS criterium in the same animal to achieve the minimum of four present DLS criteria. While this approach might be justifiable in some situations, a post-hoc augmentation of any read-out should be performed with extreme caution and while ensuring strict blinding of any experimenter involved, since the risk of selection and confirmation bias is high. Therefore, in most cases, we would advise authors to report an incomplete DLS and disclose missing or inconsistent findings as well as their statistical approach and reasoning in detail.

To assess the presence of a DLS, the most basic condition, that is the duration represented by sociofunctional impairment needs to have a duration of 7 days or more. The gathered data should be compared between groups using two parameters, which need to reflect a reduction in social interaction, or a more complex behavioural read-out like the z-score. In other behavioural sciences, the z-score has been successfully employed [165, 166]. For all DLS read-outs including the duration criterion, we strongly recommend using the z-score, since it provides a standardized score for the read-outs of interest, normalized for group mean and standard deviation (SD). This allows comparison between studies [165], which is the ultimate aim of the DLS framework. The *z*-test states how many SDs (σ) a single observation (X) deviates from the mean of a control group (μ). Hence, the following formula applies for each individual read-out:

$$Z-{{{{{\rm{test}}}}}} = \frac{{X - \mu }}{\sigma }$$

The singular z-test values, which represent a single read-out each, then need to be corrected for directionality, so that an increased score will reflect the increase of interest i.e., sociofunctional impairment. To calculate the final z-score for social interaction impairment, the individual z-scores of the read-outs reflecting social impairment need to be added up and divided by the number of tests, as below [165]:

$$Z-{{{{{\rm{score}}}}}} = \frac{{Z{{{{{\rm{test1}}}}}} + Z{{{{{\rm{test2}}}}}} + Z{{{{{\rm{test3}}}}}}}}{{{{{{{\rm{Number}}}}}}\;{{{{{\rm{of}}}}}}\;{{{{{\rm{tests}}}}}}}}$$

By using two single test read-outs or a z-score of multiple social interaction impairment proxies, statistical instruments like a repeated measures analysis of variance (ANOVA) enable the calculation of differences over the duration or per point in time. Therefore, if DLS-MESP criteria are met, the minimum duration criterion is fulfilled. Consequently, since the duration and sociofunctional impairment criteria are connected, this approach also allows one to state if the animal experienced significant sociofunctional impairment. The degree of impairment can be assessed based on the SD by comparison of the cumulative z-scores between the group of interest and the control groups. We advocate the three clinically analogous levels of severity: mild, moderate, and severe. Animals in the group of interest within one SD are mildly impaired, within the second SD are moderately impaired, and within the third SD are severely impaired. However, whether the human severity estimate is feasible for mice remains to be determined.

To determine the presence of the required biological features, we recommend using two depression-typical read-outs. Both tests per read-out need to comply with DLS-MESP criteria. However, using more than two features is likely to increase the validity of the measurements and thus DLS presence. Here, also the z-score approach could be useful to combine multiple biological read-outs and then compare the group of interest with the controls in respect to the DLS-MESP criteria.

Finally, to determine the presence of the minimum requirement of four symptoms, namely anhedonia plus three other depression-typical symptoms, we advocate using two or more behavioural tests per symptom. Both tests per symptom should meet the DLS-MESP criteria. As for the other DLS criteria, symptom assessments can either be assessed individually or be summed up into a z-score, which then can be evaluated according to the DLS-MESP criteria. Although we deem both approaches practically feasible and tenable, we suggest the use of a z-score since it enables reliable between-study comparison later on.

**Fig. 2:**
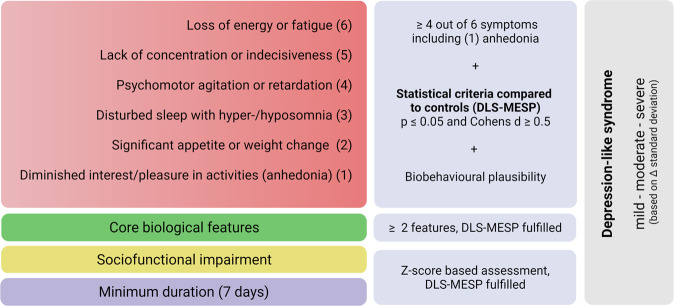
Algorithm connecting biobehavioural read-outs to DLS ascertainment. Condensed summary of the assessment process revolving around the DLS criteria. Based on the biobehavioural assessment, the algorithm allows to state if a DLS is in fact present or absent. Moreover, it enables severity estimation based on the comparison of the group of interest with controls using the standard deviation difference between the two groups’ *z*-scores (Created with BioRender.com).

In summary, we have outlined how the different biobehavioural levels can be assessed, analyzed and binarized based on the DLS-MESP criteria. This will enable researchers to clearly state the presence or absence of a DLS in mice (Fig. 2).

## Conclusion and perspectives

In summary, we herein have drafted a blueprint of a novel reference syndrome for murine depression models in translational psychiatry using defined read-out parameters based on readily available tests and methods. The DLS is a minimum threshold approach combining symptom duration and composition as well as sociofunctional impairment with core biological features. Although we have focused on a DLS approach for mice, it could be easily adjusted for laboratory rats. We believe that as a scientific community entrusted with advancing the understanding of psychiatric diseases, it is our shared obligation to consider every well-reasoned, auspicious, and ethically acceptable proposal and evaluate its strengths and weaknesses over time. A very popular example of such a proposal, admittedly on a more systemic and global level, are the 2010 published RDoC. Somewhat similar, the DLS is a work in progress, a preliminary defined murine psychosocial and biological state related to clinical depression that warrants constant testing and validation by data collection, interdisciplinary synopsis, and step-by-step optimization. The long-term objective of the DLS is to develop a between-model validation standard to improve the widespread use of imprecise phenotyping language and methodology in preclinical models of depression. We believe that an evidence-based DLS definition can provide a microframework for depression research, thus fostering the translation of clinical and preclinical findings. In the future, this DLS microframework can be further improved by the so-called ‘evo-mecho’ approach, which was recently developed by Taborsky et al. 2021. It is a comprehensive research programme connecting evolutionary modelling to empirical research, which will advance our understanding of the stress response evolution based on species and context [167]. The respective findings could help refine the DLS hypothesis and criteria of a non-random, maladaptive biobehavioural stress response pattern that is, at its very core, conserved between certain higher mammals.

The proposed syndromic DLS approach is an auspicious and labour-intensive venture and, for now, relies primarily on old fashioned behavioural assessments, which base complex phenotypes on rather simple but well-established tests like sucrose preference or social avoidance. For that reason, we propose the use of combined and normalized measure like the z-score, since this enables inter-study comparability and complex assessments, while maintaining the use of established methods and knowledge. We believe, that a DLS blueprint based on these tests might increase feasibility and acceptance, and thus aid the transformation towards consensus-based phenotyping. This is pivotal to link existing evidence based on the established tests to a DLS-based assessment and framework. Advanced phenotyping tools like the Social Box Paradigm, SimBA package, IntelliCage [168] or PsyCo [169] along with advanced facial expression tracking are gaining ground [170]. In the future, these methods could help overcome most constraints of conventional testing, foster complex assessments and read-outs, and ultimately feedback into DLS criteria. Given the purely translational hypothesis and empiric foundation of the DLS blueprint, it should prove a valuable addition to the methodological toolbox at the intersection of clinical and animal research in neuropsychiatry. Moreover, it could improve between-species comparability, aid translatability by advancing phenotypic profiling and increase overall quality of the current murine depression models. In addition, a consensus like the DLS introduces the syndrome component to animal modelling, a clinical axiom and core component of human depression widely neglected in preclinical and translational neuropsychiatry. The DLS is a longitudinal, biobehavioural definition designed to mirror and capture a syndromic state. It is not a risk or endophenotype model. In addition to the ever-necessary control group of an experimental set up, the DLS could also serve as a standardized validation option and replication platform for animal models with a clinical objective, while increasing construct and face validity.

Arguably, certain research questions might not require the syndrome concept to produce valuable findings. However, many others and especially clinically oriented translational studies might benefit from considering this clinical and human feature of depression. The DLS could fill the gap between RDoC and clinical questions of applicability and increase model scope. We urge the scientific community to consider the potential of a valid, reliable, and reproducible syndrome definition and phenotyping standard for murine depression models.
